# Socio-demographic determinants of infectious disease knowledge in Armenia

**DOI:** 10.1093/eurpub/ckac129.652

**Published:** 2022-10-25

**Authors:** Z Sargsyan, Z Grigoryan, S Sahakyan, K Kelenjian, V Hayrumyan, A Agopian, T Harutyunyan

**Affiliations:** Turpanjian College of Health Sciences, American University of Armenia, Yerevan, Armenia

## Abstract

**Background:**

There is substantial evidence that infectious disease knowledge (IDK) predicts people's behavioral intentions and preventive practices. Since level of IDK varies across socio-economic contexts and imposes a substantial burden on vulnerable groups, we aimed to assess the relationship between socio-demographic factors and IDK in the adult population of Armenia.

**Methods:**

A cross-sectional nationwide phone survey was conducted in the capital Yerevan and all Armenian provinces in 2021, using a stratified two-stage cluster sampling to complete a sample of 3,483 respondents. The questions on socio-demographic characteristics and IDK were included in a multi-domain structured survey questionnaire. Four questions measured IDK; a summative IDK score (0-4) was used in bivariate and multivariate linear regression analysis.

**Results:**

Females constituted 71.0% of the sample. The mean age was 49.5 years. About 68% of the study participants had some vocational (12-13 years) or university degree education and 54.4% were employed. About one fifth of the respondents reported family monthly expenditures of less than 100,000 Armenian drams (AMD) ≈ $200, while the majority reported spending 101,000AMD to 400,000 AMD per month. The mean IDK score was 2.48. In the adjusted analysis, being female, holding a higher education level, being employed, having younger age and higher family monthly expenditures were positively associated with IDK score.

**Conclusions:**

Our findings suggest that there is a gap in IDK affecting specific population groups such as older people, those with incomplete or secondary education, unemployed and financially disadvantaged people. Educational interventions and campaigns should target these groups to minimize the gap and ensure even prerequisites for good health.

**Key messages:**

• Gender, age, education level, employment status and income level all independently influence population’s infectious disease knowledge.

• Health communication campaigns on infectious disease knowledge should particularly target males, older people and socially disadvantaged groups.

